# Population-level genomic surveillance of human norovirus using wastewater-based whole-genome sequencing

**DOI:** 10.1128/aem.00760-26

**Published:** 2026-09-11

**Authors:** Jeongeun Mun, Chi-Hwan Choi, Minji Lee, Sun-Ju Cho, Jungwook Park, Seung Hun Lee, Sung soon Shim, Jeongjin Park, Woojin Jun, Yoon-Seok Chung

**Affiliations:** 1 Division of Emerging Infectious Disease, Department of Infectious Disease Research, Health and Environment Research Institute of Gwangju 505935 , Gwangju, Republic of Korea; 2 Division of High-risk Pathogens, Department of Laboratory Diagnosis and Analysis, Korea Disease Control and Prevention Agency 204189, Cheongju, Republic of Korea; 3 Division of Food and Nutrition, Chonnam National University 98413, Gwangju, Republic of Korea; 4 Research Institute for Human Ecology, Chonnam National University 294166, Gwangju, Republic of Korea; Centers for Disease Control and Prevention, Atlanta, Georgia, USA

**Keywords:** wastewater-based surveillance, human norovirus, whole-genome sequencing, quasispecies, genotype dynamics

## Abstract

Wastewater-based surveillance has garnered increasing attention as a valuable approach for capturing community-level infection dynamics that are often difficult to detect through clinical reporting systems alone. In this study, we analyzed human norovirus genotype distributions and whole-genome-level variations in wastewater samples collected in Gwangju, Korea. These results were interpreted in conjunction with a documented foodborne outbreak to evaluate the epidemiological relevance of wastewater-based monitoring. Human norovirus concentrations were quantified using TaqMan Array Card–based RT-qPCR, and whole-genome next-generation sequencing (NGS) was performed to obtain viral read counts and reads per kilobase per million filtered reads values. Overall, strong correlations were observed between RT-qPCR-based concentrations and NGS-derived metrics. Genotype dynamics varied among wastewater treatment plants, reflecting differences in catchment size and local population characteristics. In particular, the relative abundance of GII.17[P17] increased during epidemiological week 50, temporally coinciding with a documented local foodborne outbreak. Variant analysis revealed that wastewater samples exhibited mixed nucleotide patterns, with multiple alleles coexisting at varying relative frequencies rather than fixed substitutions. Notably, some nonsynonymous variants detected in clinical samples were also observed in wastewater samples collected surrounding the outbreak period. Together, these findings demonstrate that wastewater-based whole-genome surveillance can capture both genotype-level shifts and nucleotide-level dynamics at the population scale, highlighting its potential as a complementary tool for monitoring community-level norovirus circulation and outbreak-associated genotype dynamics.

**IMPORTANCE:**

Wastewater-based surveillance is increasingly recognized as a promising approach for capturing community-level infection dynamics that are often missed by clinical surveillance. In this study, we applied whole-genome sequencing to wastewater samples collected in Gwangju, South Korea, to comprehensively characterize human norovirus genotype distributions and genetic variation. Distinct genotype patterns were observed across wastewater treatment plants, reflecting differences in catchment population size and local characteristics. Notably, an increase in the GII.17[P17] genotype detected in wastewater coincided with a foodborne outbreak investigated in Gwangju, demonstrating the potential of wastewater surveillance to reflect ongoing community transmission and emerging outbreak-associated genotypes. In addition, wastewater samples contained diverse and coexisting genetic variants, capturing population-level viral diversity and evolutionary dynamics that are not readily detected through clinical surveillance alone. These findings highlight the value of wastewater-based whole-genome surveillance for monitoring community-level viral circulation and support its integration as a complementary strategy to existing clinical surveillance systems.

## INTRODUCTION

Human norovirus is a leading cause of acute gastroenteritis worldwide and accounts for a substantial proportion of foodborne outbreaks (1). Ongoing circulation and frequent outbreak events have been reported across multiple countries, underscoring the continued public health significance of human norovirus infections (2, 3). In Korea, human norovirus is consistently identified as the most frequently detected enteric virus, and surveillance data from the Korea Disease Control and Prevention Agency (KDCA) Enter-Net program, together with statistics from the Ministry of Food and Drug Safety, indicate that it remains a major etiological agent of foodborne illness (4, 5).

Most infectious disease surveillance systems rely on clinical reporting based on RT-PCR testing of stool samples obtained from individuals who seek medical care (6, 7). While effective for case confirmation, this approach likely underestimates the true burden of infection because it fails to adequately capture asymptomatic or mildly symptomatic individuals. Previous studies have estimated that approximately 21.8% of human norovirus infections are asymptomatic, and that asymptomatic and symptomatic individuals shed comparable viral loads for similar durations (8, 9). Consequently, failure to account for asymptomatic infections may bias interpretations of community-level transmission dynamics.

To address these limitations, wastewater-based surveillance has gained increasing attention as a complementary population-level monitoring strategy that integrates infection signals from the entire community, regardless of symptom status or healthcare-seeking behavior. Because wastewater constitutes a non-selective matrix that reflects aggregated viral shedding, it enables sensitive and unbiased monitoring of pathogen circulation at the community scale. Human norovirus is particularly well suited to this approach, as it is primarily excreted in feces and vomitus, is shed in high concentrations during the early phase of infection, and can be detected even prior to symptom onset (10, 11). Despite these advantages, wastewater samples present substantial technical challenges for genomic analysis due to the presence of complex microbial communities and environmental contaminants. In this context, whole-genome next-generation sequencing (NGS) has become an essential tool for the high-resolution characterization of viral genomes of public health importance. Given the extensive genetic diversity and rapid evolutionary dynamics of human norovirus, whole-genome sequencing is necessary not only to discriminate among genotypes but also to resolve intra-genotypic variation and recombination events that cannot be captured by partial genomic approaches. In this study, we conducted whole-genome sequencing of human norovirus in wastewater samples collected from weeks 36 to 52 of 2024, alongside clinical samples obtained from outbreak-associated cases. Our objective was to characterize temporal changes in human norovirus genotypes and genetic variation using wastewater-based surveillance combined with whole-genome sequencing, and to assess the utility of this integrated approach for elucidating community-level infection dynamics.

## MATERIALS AND METHODS

### Wastewater sample collection

Influent wastewater samples were collected from three major wastewater treatment plants (WWTPs) in Gwangju, Republic of Korea, namely Gwangju WWTP No. 1 (Site 1), Gwangju WWTP No. 2 (Site 2), and Hyocheon WWTP (Site 3), from weeks 36 to 52 of 2024. These WWTPs collectively serve nearly the entire population of the city and represent distinct catchment areas. Site 1 is located in Chipyeong-dong, Seo-gu, in the western part of Gwangju metropolitan city, and serves four of the city’s five administrative districts, covering approximately 83% of the total population. Site 2 is located in Bondeok-dong, Gwangsan-gu, in the southwestern part of the city, and serves one administrative district, representing approximately 16% of the total population. Site 3 is located in Imam-dong, Nam-gu, in the southern part of Gwangju and serves the Jinwol area, Hyocheon 1 and 2 residential development areas, Bitgoeul Senior Welfare Town, Gwangju University, and nearby rural villages, representing approximately 1% of the total population. Approximately 1 L of influent wastewater was collected weekly from each WWTP with the assistance of the Gwangju Environment Corporation. Samples were obtained by direct grab sampling from the incoming untreated wastewater stream prior to treatment. All WWTPs have treatment capacities exceeding 10,000 m³ per day (Table 1), ensuring broad population coverage and supporting the use of wastewater surveillance as a proxy for community-level infection dynamics (12).

**TABLE 1:** Characteristics of wastewater treatment plants

Characteristic	WWTP		
	Site 1	Site 2	Site 3
Treatment capacity (m^3^/day)	600,000	120,000	16,000
Population served	1,160,000	190,000	50,000
Sampling method	Direct sampling	Direct sampling	Direct sampling
Sampling frequency	Weekly	Weekly	Weekly

### Nucleic acid extraction and TaqMan array card

For each sampling event, a 1-L grab influent wastewater sample was collected from each wastewater treatment plant and divided into two 15-mL aliquots. Each aliquot was transferred into a 50-mL conical tube and centrifuged at 3,000 × *g* for 30 min (5810R, Eppendorf, Germany) to remove suspended solids and particulate matter. *Bacillus atrophaeus* was added to each aliquot as an extraction control prior to nucleic acid extraction. Subsequently, 10 mL of the clarified supernatant was used for viral RNA extraction using an automated extraction system (Chemagic 360, PerkinElmer, USA) with the Viral DNA/RNA 10K Kit (#CMG-749), according to the manufacturer’s instructions. Only samples with an extraction recovery efficiency of ≥10% were included in subsequent analyses. Prior to TAC loading, a pre-amplification step was performed using a ProFlex PCR System (Applied Biosystems, Thermo Fisher Scientific, Waltham, MA, USA). The customized TaqMan Array Card (TAC; Thermo Fisher Scientific, Waltham, MA, USA) is a microfluidic real-time PCR platform for simultaneous detection of multiple pathogens. The 10-μL reaction mixture consisted of 2.5 μL of TaqPath 1-Step RT-qPCR Master Mix (4×) (Thermo Fisher Scientific, Waltham, MA, USA), 2.5 μL of a custom target-specific primer pool designed for the customized TaqMan Array Card, and 5 μL of extracted RNA. The customized TaqMan Array Card included assays for human norovirus GI (Assay ID: Vi07922261_po) and GII (Assay ID: Vi07922262_po); however, only GII concentrations were used for comparison with NGS-derived metrics because sequencing analyses were performed exclusively for norovirus GII. The pre-amplification program consisted of reverse transcription at 50°C for 30 min, enzyme activation at 95°C for 2 min, followed by 14 cycles of 95°C for 15 s and 60°C for 2 min. Each TAC port was loaded with 100 μL of reaction mixture consisting of 10 μL of the pre-amplified product, 50 μL of TaqMan Fast Advanced Master Mix (Thermo Fisher Scientific, Waltham, MA, USA), and 40 μL of nuclease-free water. The loaded card was centrifuged at 1,000 × *g* for 1 min, sealed, and analyzed using a QuantStudio 12K Flex Real-Time PCR System (Applied Biosystems, Thermo Fisher Scientific, Waltham, MA, USA). The thermal cycling program consisted of an initial activation at 95°C for 10 min, followed by 40 cycles of 95°C for 3 s and 60°C for 30 s. Quantification was performed using calibration curves generated from reference standards provided by the Korea Disease Control and Prevention Agency, and a no-template control (nuclease-free water) was included in each run.

### NGS library preparation and whole-genome sequencing

Extracted RNA from clinical and wastewater samples (9 μL per sample) was reverse-transcribed into complementary DNA (cDNA) using the SuperScript IV First-Strand Synthesis System (Thermo Fisher Scientific, Waltham, MA, USA), according to the manufacturer’s instructions. The synthesized cDNA was subsequently quantified, and 100 ng of cDNA (17.5 μL) was used as the input for library preparation. The resulting cDNA was subjected to library preparation using two different sequencing approaches. For wastewater samples from Sites 1, 2, and 3 collected during the 2024 epidemiological weeks (EWs) 36–52, libraries were prepared using a hybrid-capture–based enrichment approach with the Illumina Viral Surveillance Panel (VSP; Illumina, San Diego, CA, USA), followed by sequencing on the Illumina NovaSeq 6000 platform (Illumina, San Diego, CA, USA). For a subset of wastewater samples from Sites 1, 2, and 3 collected during EWs 38 and 39 and 48–50, primer-based amplicon sequencing was performed using the Genexus Integrated Sequencer (Thermo Fisher Scientific, Waltham, MA, USA). Libraries were generated using the AmpliSeq RNA GX Panel (A47577) with Genexus Primer Pool Tubes (A40262), Library Strips 1 and 2-AS (A40252), and Barcodes 1–32 AS (A40258), according to the manufacturer’s instructions. The entire workflow, from cDNA synthesis through sequencing on the Genexus system, was fully automated.

### Bioinformatic processing, genotyping, and variant analysis

Sequencing reads generated from both the Illumina NovaSeq 6000 platform (paired-end) and the Genexus system (single-end) were processed using the EsViritu (v1.1.1) workflow to remove PhiX control reads and host-derived sequences from Homo sapiens. Filtered reads were subsequently mapped to a viral reference database (esviritu_db_v3.2.2) to identify appropriate reference sequences and generate human norovirus consensus genomes. For clinical–wastewater comparisons at Site 1 (EWs 38 and 39 and 48–50), Illumina hybrid-capture sequencing data from clinical samples were paired with Genexus amplicon sequencing data from wastewater samples. This approach was adopted because hybrid-capture sequencing of wastewater samples yielded insufficient sequencing depth for robust variant analysis during these epidemiological weeks. Primer sequences for the Genexus amplicon assay were not provided by Thermo Fisher Scientific.

Human norovirus consensus sequences were genotyped using the Human Calicivirus Typing Tool (https://calicivirustypingtool.cdc.gov/bctyping.html). Variant calling was performed using CLC Genomics Workbench v25.0.2 (CGW) with a GII.17[P17] reference sequence (GenBank accession PQ310520), selected based on the clinical genotype. Variant positions were defined relative to the corresponding clinical consensus sequence to enable direct comparison with wastewater-derived consensus sequences. All human norovirus consensus sequences were aligned to the same GII.17[P17] reference sequence (PQ310520) using Nextalign. The aligned genomes were visualized using SNIPIT, with positions of interest (POIs) identified from CGW variant calls supplied as an input list to highlight variants of interest. Manual curation was performed to assess within-sample minority variant proportions characteristic of quasispecies.

### Outbreak investigation and clinical specimen collection

A suspected foodborne outbreak occurred at a group catering facility in Gwangju in December 2024, involving 35 individuals with gastrointestinal symptoms. Of the 35 symptomatic individuals, rectal swab specimens were collected from 22 patients who agreed to specimen collection and were included in the laboratory investigation. The swabs were immersed in 5 mL of phosphate-buffered saline and homogenized using a vortex mixer for approximately 3 min to release viral particles. The specimens were screened for 29 foodborne pathogens, including bacterial, viral, and protozoan agents, according to the standard diagnostic workflow, using bacterial culture, PCR-based molecular assays, and other appropriate diagnostic methods.

### Norovirus RNA extraction and genotyping

Following routine diagnostic testing, residual specimens that tested positive for human norovirus were used for molecular characterization in this study. Viral RNA was extracted using the QIAamp Viral RNA Mini Kit (Qiagen, Hilden, Germany) according to the manufacturer’s instructions. The human norovirus genome was amplified using the SB-Plex human norovirus GII Sequencing Kit (Seegene, Seoul, Korea) with the primer pair GII-MON431 and GII-G2SKR. Genotypes were assigned using the human norovirus Genotyping Tool (RIVM, the Netherlands; http://www.rivm.nl/mpf/norovirus/typingtool) according to the dual-typing system.

## RESULTS

### Association between quantitative human norovirus load and NGS-based metrics

**Fig 1:**
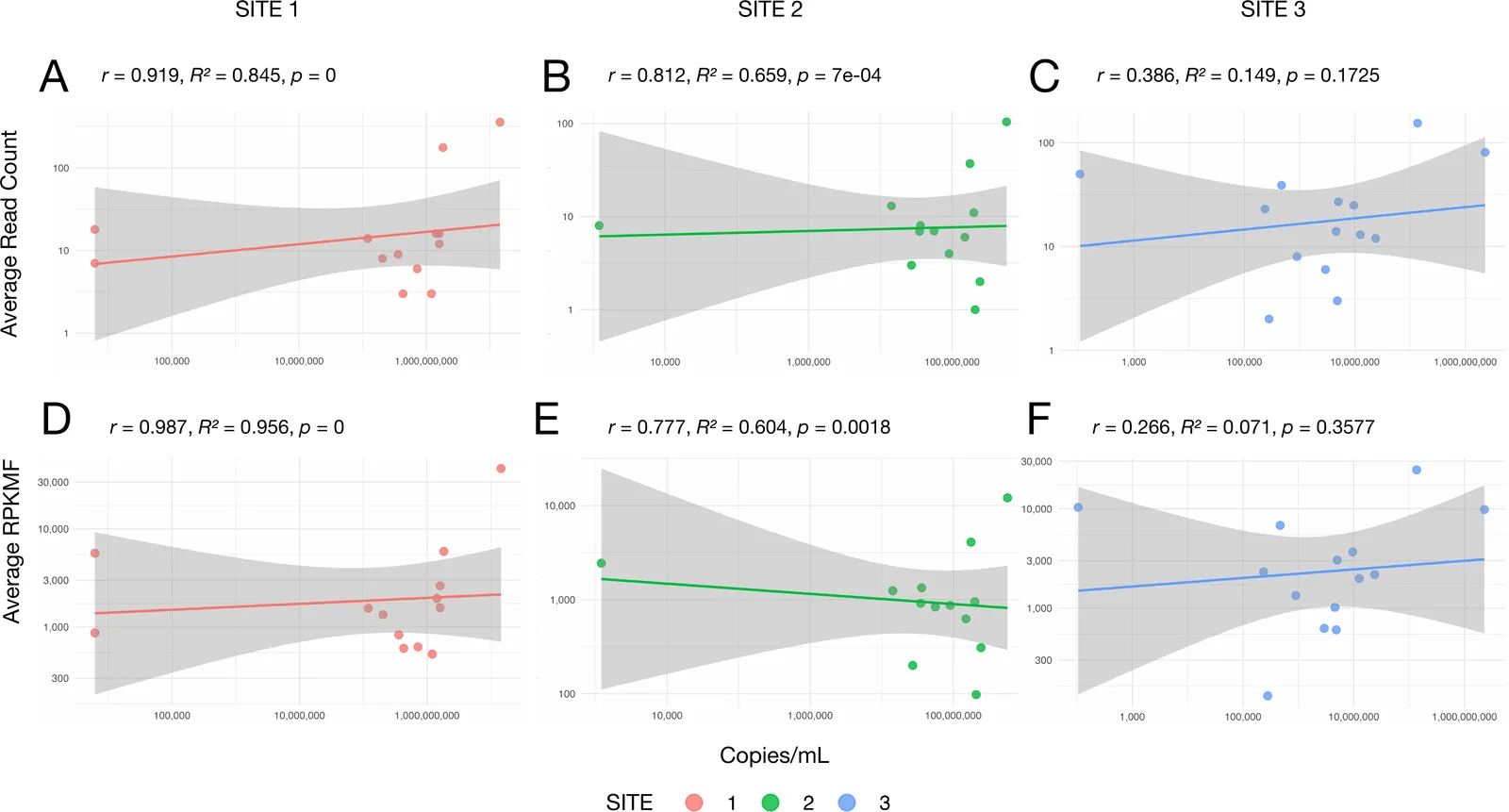
Correlations between norovirus GII copies/mL and NGS metrics at three wastewater sampling sites. (**A–C**) Average read counts at Sites 1–3, respectively; (**D–F**) average RPKMF values at Sites 1–3, respectively.

A correlation analysis was performed to evaluate the relationship between human norovirus concentrations (copies/mL) and NGS-derived metrics, including reads per kilobase per million filtered reads (RPKMF) and read counts, in wastewater samples. When analyzed separately, both Site 1 and Site 2 showed strong positive correlations between human norovirus concentrations and NGS-derived metrics, although the correlations were stronger at Site 1. For read counts, the correlation coefficients were *R* = 0.919 (Site 1) and *R* = 0.812 (Site 2) (Fig. 1A and B). For RPKMF, the corresponding values were *R* = 0.987 (Site 1) and *R* = 0.777 (Site 2) (Fig. 1D and E). In contrast, weaker correlations were observed at Site 3. Specifically, the correlation between human norovirus concentration and read counts was relatively low (*R* = 0.386; Fig. 1C), and a similarly weak association was detected between human norovirus concentration and RPKMF (*R* = 0.266; Fig. 1F).

### Temporal variation of predominant human norovirus GII genotypes

**Fig 2:**
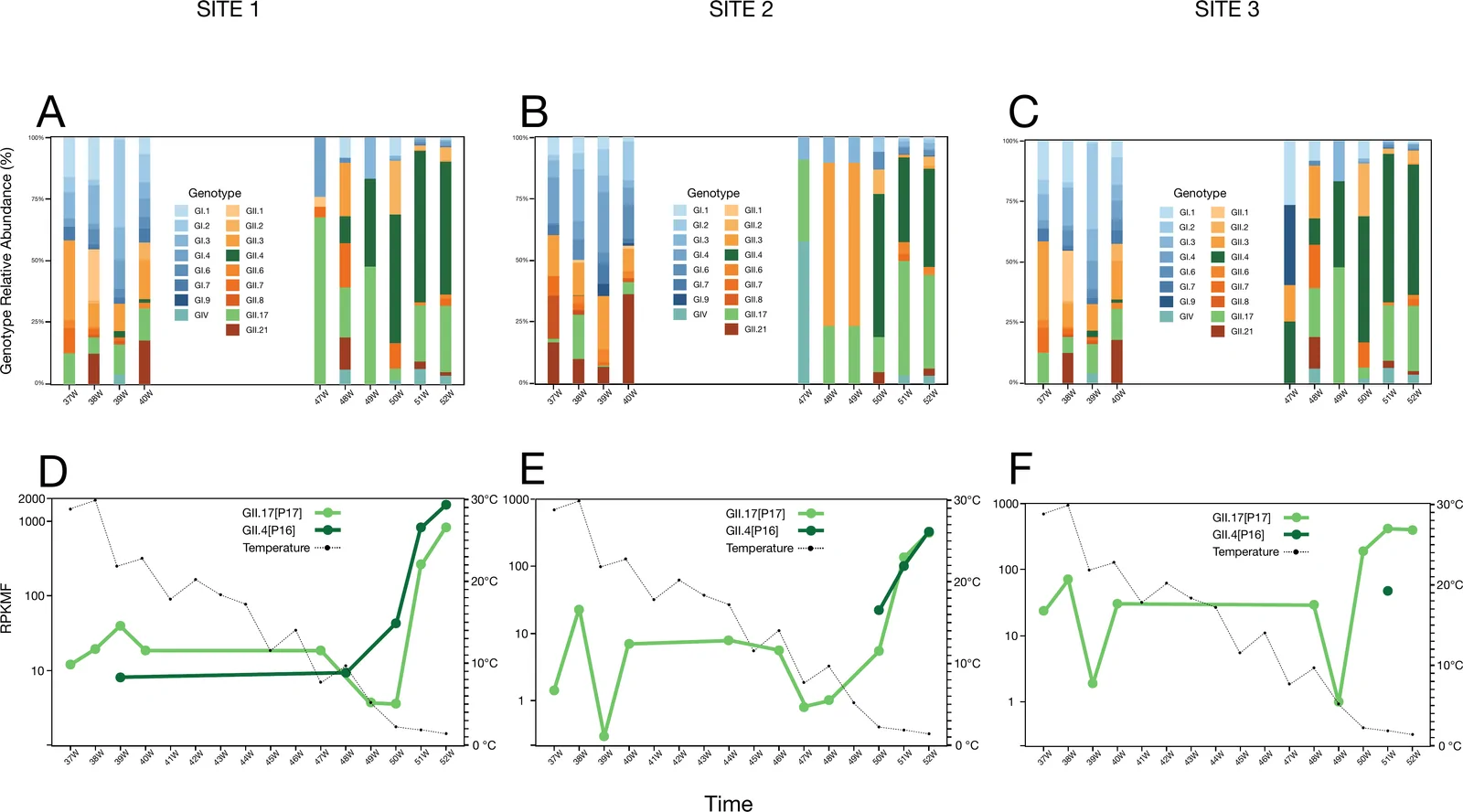
Temporal trends of predominant norovirus GII genotypes based on NGS-derived RPKMF values across three wastewater treatment plants. (**A–C**) Relative abundance of each norovirus genotype, estimated from NGS-derived RPKMF values, is shown for each sampling time point. Relative abundance was calculated by normalizing the RPKMF value of each genotype to the total RPKMF of all detected norovirus GII genotypes within each sample. (**D–F**) Raw NGS-derived RPKMF values of the predominant genotypes, GII.4[P16] and GII.17[P17], are shown together with ambient temperature during the sampling period.

The relative abundance of human norovirus genotypes was estimated for each wastewater treatment plant based on RPKMF values derived from NGS data. In addition, the raw RPKMF values of the predominant genotypes were presented to illustrate temporal changes in genotype-specific abundance. Although multiple genogroups, including GI, GII, and GIV, were simultaneously detected in wastewater samples, GII.4[P16] and GII.17[P17] were identified as the predominant genotypes and were therefore selected as the primary focus of the temporal pattern analysis. At Site 1, the relative abundance of both GII.17[P17] and GII.4[P16] increased after week 50 (Fig. 2A). At Site 2, GII.17[P17] similarly showed an increased relative abundance after week 50, while GII.4[P16] also accounted for a substantial proportion of the genotype composition during the same period (Fig. 2B). At Site 3, GII.17[P17] also exhibited an increased relative abundance after week 50 (Fig. 2C). The raw RPKMF values showed temporal trends consistent with the relative abundance analysis, with increased RPKMF values for GII.17[P17] after week 50 across all three sites and for GII.4[P16] at Sites 1 and 2 (Fig. 2D through F). Notably, GII.17[P17] was continuously detected across all three wastewater treatment plants throughout the study period and exhibited increased relative abundance and RPKMF values after week 50.

### Human norovirus detection and genotyping in a suspected foodborne outbreak

Among the 22 rectal swab specimens collected during the outbreak investigation, human norovirus GII was detected in 16 specimens. Genotype analysis revealed that 12 of the norovirus-positive specimens were identified as GII.17[P17], while the remaining four specimens could not be assigned to a specific genotype (Table 2).

**TABLE 2:** Summary of human norovirus detection and genotype distribution in human rectal swab specimens collected during a suspected foodborne outbreak in Gwangju

Category	No. of samples	Human norovirus-positive samples	Detected genotype
Human	22	16	GII.17[P17] (*n* = 12)
			Untypable (*n* = 4)

### Temporal patterns of nucleotide variation in clinical and wastewater-derived human norovirus GII.17[P17]

**Fig 3:**
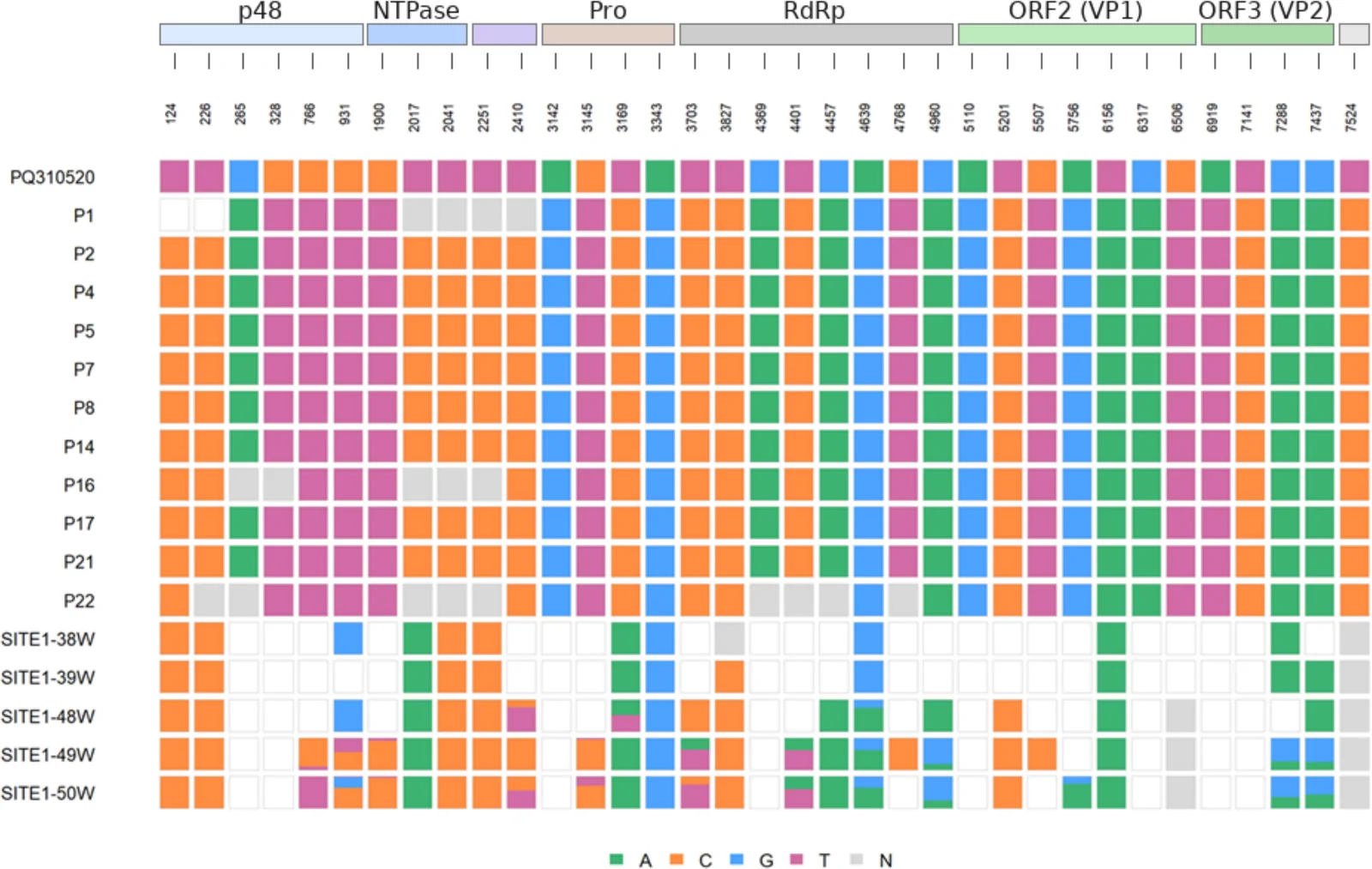
Single-nucleotide polymorphisms (SNPs) identified by comparison with the reference genome PQ310520. Colored cells indicate nucleotide variants (A, C, G, and T), whereas blank cells represent positions identical to the reference sequence. The clinical genome was obtained during the epidemiologically defined outbreak period (EW 50). Wastewater samples represent available specimens collected at selected epidemiological weeks (EWs 38 and 39 and EWs 48–50) surrounding the outbreak and are displayed in chronological order. For wastewater samples, nucleotide compositions are displayed as relative frequencies at each genomic position across sampling weeks 38, 39, 48, 49, and 50, reflecting the presence of mixed viral populations. Positions with insufficient sequencing depth (<10× ) are labeled as “N.”

Using the GII.17[P17] strain PQ310520 as the reference sequence, nucleotide variations in clinical specimens were analyzed. Although all clinical samples belonged to the same genotype, multiple nucleotide differences relative to the reference sequence were observed. In total, 35 single-nucleotide polymorphisms (SNPs) were identified. Of these, three were classified as nonsynonymous substitutions, located at positions 4401 (I > T) and 4457 (A > T) in ORF1 and at position 6156 (F > I) in ORF2 (VP1), while the remaining substitutions were synonymous. The 35 variant positions were defined based on sites consistently detected in the clinical specimens, and these same positions were subsequently used for comparative analysis of wastewater samples. Among the 12 outbreak-associated clinical genomes initially available for SNP analysis, one genome was excluded because its horizontal genome coverage was insufficient for reliable nucleotide variant analysis. Consequently, SNP analysis was performed using the remaining 11 clinical genomes collected during epidemiological week 50. Wastewater samples used for comparison were collected from Site 1 during epidemiological weeks 38, 39, 48, 49, and 50. Positions labeled as “N” indicate regions with insufficient sequencing depth (<10× ), where reliable base calling was not possible. In wastewater samples, nucleotides were not fixed to a single base but instead occurred as mixtures with varying relative proportions at each position. Accordingly, wastewater-derived variants were represented by their relative nucleotide frequencies rather than as discrete substitutions. Comparison of weekly nucleotide distributions (weeks 38–50) for the nonsynonymous substitutions revealed distinct temporal patterns across positions. At position 4401, the nucleotide identical to the reference sequence remained dominant throughout most weeks, with mixed patterns observed only at a limited number of time points. In contrast, at position 4457, the reference nucleotide predominated during the early weeks, whereas the nucleotide observed in the clinical specimens became more prevalent or appeared as a mixed pattern during later weeks. At position 6156, the same nucleotide detected in the clinical specimens was consistently observed across all wastewater sampling weeks. Overall, wastewater samples exhibited dynamic temporal changes in the relative proportions of reference and alternative nucleotides at individual positions (Fig. 3).

## DISCUSSION

Wastewater samples are inherently variable in quantitative viral analyses due to the presence of PCR inhibitors, RNA degradation, dilution effects caused by fluctuations in influent flow rates, and the coexistence of diverse nucleic acids (13). These factors may also introduce uncertainty into NGS-based analyses, including assessments of genotype diversity and recombination. Consistent with these inherent sources of variability, the strength of the correlation between RT-qPCR concentrations and NGS-derived metrics varied among the three WWTPs. Nevertheless, when data from all sites were considered collectively, RT-qPCR measurements obtained using the TaqMan Array Card platform showed a strong overall correlation (*R* ≈ 0.9) with NGS-derived abundance metrics, including viral read counts and RPKMF values. These findings suggest that, despite site-specific variability, NGS-derived abundance metrics provide a reasonable proxy for relative temporal changes in norovirus concentrations in wastewater. Furthermore, these findings support the complementary use of NGS for wastewater surveillance, as sequencing provides genotype-specific information while reflecting temporal changes in viral abundance.

The correlation between RT-qPCR concentrations and NGS-derived abundance metrics was weaker at Site 3 than at Sites 1 and 2. Site 3 (Hyocheon WWTP) has a substantially smaller treatment capacity (16,000 m³/day) and serves approximately 50,000 people, whereas Site 1 (Gwangju WWTP No. 1) and Site 2 (Gwangju WWTP No. 2) have treatment capacities of 600,000 and 120,000 m³/day and serve approximately 1.16 million and 190,000 people, respectively. Because Site 1 serves the largest catchment population in Gwangju, wastewater collected at this facility may provide a more integrated representation of community-wide viral shedding than localized transmission events (14). The viral concentrations and genotype distributions observed at Site 1 may therefore better reflect broader community-level norovirus circulation. Site 2 also showed relatively strong correlations between RT-qPCR concentrations and NGS-derived abundance metrics, despite serving a smaller catchment population than Site 1. Previous studies have identified catchment population, sewer system characteristics, and wastewater flow dynamics as important sources of variability and uncertainty in wastewater-based epidemiology (15). In contrast, the relatively small catchment served by Site 3 may be more susceptible to localized fluctuations in viral shedding, which may have contributed to the weaker correlation observed in this study. However, because the influence of catchment characteristics on measurement variability was not directly evaluated, this interpretation should be regarded as a plausible explanation rather than a definitive conclusion.

Across all WWTPs, shifts in human norovirus genotype proportions were observed around epidemiological week 50, although the direction and magnitude of these changes varied by site. Despite site-specific differences, genotype relative abundance consistently indicated an increase in GII.17[P17] after week 50 across all three WWTPs. This trend was similarly reflected by the raw RPKMF values, indicating consistent temporal patterns between genotype relative abundance and raw RPKMF measurements. Notably, GII.17[P17] was continuously detected throughout the study period at all three WWTPs, suggesting that this genotype had already been circulating within the community prior to the outbreak. Its relative abundance and raw RPKMF values increased after week 50, coinciding with the outbreak period. The foodborne outbreak occurred the week 50 wastewater sampling events, and the same GII.17[P17] genotype was identified in rectal swab specimens collected from outbreak-associated patients. During a similar period, South Korea reported a marked increase in GII.17[P17] beginning in November 2024, with this genotype rapidly replacing GII.4-associated strains to become the predominant genotype within a single winter season (16). A similar epidemiological trend has also been observed internationally. During the 2023/2024 norovirus season, several European countries and the United States reported increased GII.17-associated outbreaks and sporadic infections, with GII.17 accounting for 17%–64% of all GII detections while the prevalence of GII.4 declined (17). Furthermore, a marked resurgence of GII.17[P17] was reported in China during the 2024–2025 season, indicating continued global expansion of this genotype (18). Taken together, these findings suggest that the temporal increase in GII.17[P17] observed in Gwangju was consistent with the nationwide genotype replacement in South Korea and concurrent international reports of GII.17[P17] resurgence.

Although the outbreak could not be directly linked to a specific WWTP because the detailed catchment boundaries of the treatment plants were not available, the continuous detection of GII.17[P17] in wastewater prior to the outbreak, together with its increased relative abundance during the outbreak period, suggests that the outbreak represented a clinical manifestation of a genotype that had already been circulating in the community. These findings indicate that wastewater-based surveillance can provide valuable information on temporal changes in circulating norovirus genotypes and their population-level circulation patterns beyond reflecting individual outbreak events (19). To further characterize these genomic changes, outbreak-associated clinical and wastewater genomes were compared at the nucleotide level. Comparison of wastewater samples using the 35 SNP positions identified in the outbreak-associated clinical specimens showed that the corresponding nucleotides in wastewater were not present as fixed substitutions but rather as mixtures of reference and alternative nucleotides with varying relative proportions over time (20). Among the three nonsynonymous SNPs, the clinical-associated nucleotide at position 6156 was consistently detected in wastewater samples throughout epidemiological weeks 38–50, whereas the proportion of the clinical-associated nucleotide at position 4457 increased during epidemiological weeks 49 and 50. Notably, several nucleotide variants identified in the outbreak-associated clinical specimens were already detectable as mixed variants in wastewater samples collected before the outbreak and were also observed in wastewater collected during the outbreak period. These findings suggest that some outbreak-associated nucleotide variants had already been present within the genetically diverse viral population circulating in the community before clinical confirmation (21). Collectively, these results demonstrate that wastewater genomic surveillance can continuously capture genetic changes in circulating norovirus populations and provide population-level genomic context for interpreting genotype and nucleotide changes identified during outbreak investigations (22). These findings extend previous wastewater-based norovirus studies, which have primarily focused on identifying circulating genotypes, monitoring genotype replacement, and, in some cases, detecting emerging epidemic strains before they are recognized through clinical surveillance (23). More recent studies have further highlighted the value of wastewater sequencing for characterizing viral genetic diversity and supporting molecular epidemiology (24). In contrast, our study further evaluated temporal mutation profiles within wastewater viral populations and compared them with mutations identified in outbreak-associated clinical genomes. Although direct comparison between pooled wastewater sequences and individual clinical genomes has inherent limitations, our findings suggest that mutation-level analysis of wastewater samples may provide complementary information to conventional genotype-based surveillance and may further enhance genomic surveillance as sequencing technologies and analytical methods continue to advance. Despite these strengths, this study has several interpretational limitations. Although week-to-week changes in variant distributions were examined through comparisons of clinical and wastewater genomes, the lack of region-specific reference genomes representing previously circulating GII.17[P17] strains in Gwangju limits our ability to determine whether the observed variants represent newly emerged mutations or pre-existing variants already circulating within the local community (25). Furthermore, environmental and methodological factors that may affect viral RNA recovery, such as actual wastewater temperature and PCR inhibitors, were not specifically monitored in this study. Consequently, their potential influence on RNA recovery efficiency and genotype detection could not be evaluated. Although all samples were processed using identical concentration, RNA extraction, and sequencing protocols to minimize technical variability, these unmeasured factors should be considered when interpreting the findings. Collectively, these findings demonstrate that wastewater-based NGS surveillance can capture not only shifts in human norovirus genotype composition but also population-level changes in nucleotide-level variants over time. Moreover, by interpreting wastewater-derived genetic signals alongside clinical surveillance data, this study demonstrates the potential of wastewater-based genomic surveillance to complement conventional surveillance and improve our understanding of community-level norovirus circulation.

### Conclusion

This study demonstrates that wastewater-based whole-genome surveillance can serve as a population-level monitoring tool for characterizing community-wide human norovirus circulation. Whole-genome NGS was successfully applied to wastewater samples, enabling characterization of genotype distributions and population-level genomic variation. The concordance between wastewater-derived genomic signals and outbreak-associated clinical genomes further demonstrates the utility of wastewater whole-genome sequencing for monitoring circulating norovirus populations at the community level. In addition, the observation of certain variants in wastewater concurrently with, and in some cases earlier than, their identification in clinical samples suggests that wastewater-based genomic surveillance may complement clinical surveillance by providing early genomic signals associated with emerging outbreaks. Overall, integrating wastewater-based surveillance with whole-genome NGS provides a high-resolution framework for monitoring circulating norovirus populations and supports its broader application as a complementary component of future infectious disease surveillance systems.

## Data Availability

All raw sequencing data generated in this study have been deposited in the NCBI Sequence Read Archive (SRA). Wastewater amplicon sequencing data generated using the Genexus platform are available under BioProject accession number PRJNA1426496. Wastewater whole-genome sequencing data generated using the Illumina NovaSeq 6000 platform are available under BioProject accession number PRJNA1426785. Clinical targeted-capture sequencing data are available under BioProject accession number PRJNA1426737.
